# Occurrence Patterns of Afrotropical Ticks (Acari: Ixodidae) in the Climate Space Are Not Correlated with Their Taxonomic Relationships

**DOI:** 10.1371/journal.pone.0036976

**Published:** 2012-05-22

**Authors:** Agustín Estrada-Peña, Adrián Estrada-Sánchez, David Estrada-Sánchez

**Affiliations:** Department of Parasitology, Faculty of Veterinary Medicine, University of Zaragoza, Zaragoza, Spain; University of Minnesota, United States of America

## Abstract

Foci of tick species occur at large spatial scales. They are intrinsically difficult to detect because the effect of geographical factors affecting conceptual influence of climate gradients. Here we use a large dataset of occurrences of ticks in the Afrotropical region to outline the main associations of those tick species with the climate space. Using a principal components reduction of monthly temperature and rainfall values over the Afrotropical region, we describe and compare the climate spaces of ticks in a gridded climate space. The dendrogram of distances among taxa according to occurrences in the climate niche is used to draw functional groups, or clusters of species with similar occurrences in the climate space, as different from morphologically derived (taxonomical) groups. We aim to further define the drivers of species richness and endemism at such a grid as well as niche similarities (climate space overlap) among species. Groups of species, as defined from morphological traits alone, are uncorrelated with functional clusters. Taxonomically related species occur separately in the climate gradients. Species belonging to the same functional group share more niche among them than with species in other functional groups. However, niche equivalency is also low for species within the same taxonomic cluster. Thus, taxa evolving from the same lineage tend to maximize the occupancy of the climate space and avoid overlaps with other species of the same taxonomic group. Richness values are drawn across the gradient of seasonal variation of temperature, higher values observed in a portion of the climate space with low thermal seasonality. Richness and endemism values are weakly correlated with mean values of temperature and rainfall. The most parsimonious explanation for the different taxonomic groups that exhibit common patterns of climate space subdivision is that they have a shared biogeographic history acting over a group of ancestrally co-distributed organisms.

## Introduction

Factors that affect the life cycle of parasitic arthropods, like ticks, have been proposed as possible limiting factors for their ranges, and include host availability [1], [2] vegetation type and structure [3] and climate [4], [5]. While these variables are all closely interrelated, both statistically and biologically, their relative importances differ. Most of the ticks in the Afrotropical region for which reasonable numbers of collection records exist do not show high levels of host specificity, and few (if any) of these species can be considered host-limited [6], [7]. Biotic variables such as vegetation type and host distributions, may be important in creating heterogeneity in tick distributions at smaller scales [3], but play a subordinate role in limiting the species ranges of ticks. Cumming [8] determined the most important broad-scale variables as range determinants of ticks and concluded that their geographical range in Africa is largely determined by the direct effects of climate.

Differences in niches for tick taxa are quantified using observed occurrences of species and reflect a yet unknown conjunction of the environmental space of the species, the biotic interactions they experience and the habitats available to species and colonized by them [5]. Many species may co-occur in an area, creating the setting for an array of interactions with cascading direct and indirect effects [4]. Documenting these interactions, even for a subset of species, is an overwhelming challenge, especially as the nature of the interactions and their effects on any one species may change from place to place as other species enter or leave an assemblage or their abundances change [8]. To define how ticks occupy a geographical range, we need first to characterize their preferences in the climate (not geographical) niche. Information come from widely used, on-line collections of climate data such as WorldClim [9], or remotely sensed climate features [10] together with a wealth of reliably information on geographically referenced records. Temperature, water vapor contents of air and photoperiod (for those species with dependance upon a number of light hours) can produce a strict definition of the habitat (e.g. Ref [5]), Other variables may be only used as categorical classifications (e.g. vegetation types) or are reasonably correlated with pure climate features (e.g. temperature and altitude) therefore lacking further interest because the introduction of redundant information.

Recent concerns over the impacts of climate trends on the distribution of ticks [11], [12] have emphasized the need to quantify differences in climate requirements among species. There is also a growing interest on the paleophylogeography, this is the evaluation and computation of ancestral climate envelopes of groups of species (e.g. Ref [13]). These procedures compare the phylogenetic relationships of the organisms, based on the fossil record or genetic distances based on DNA sequences, comparing them with the presumed common, ancestral climate envelope of the whole set of considered taxa. It is thus of interest to characterize the extent of species and the explicit associations of taxonomical groups (previously made only on a morphological approach) to precise portions of the climate space.

This paper is aimed to define the climate envelope for tick species recorded in the Afrotropical region and the relationships between groups of species, without explicit consideration of the geographical space. The study is not focused around the range of the ticks in the geographical space, but in the climate one. We describe and compare climate envelopes of ticks recorded in the Afrotropical region in a gridded climate space (i.e. where each cell corresponds to a unique set of environmental conditions) and we aim to define features of species richness and endemism at such a grid. We thus explicitly sought to describe the relationships among morphologically recognized taxa according to their strict positions in the climate niche and to consider how diversity of available niches may relate to speciation and divergence from a common pool of lineages. This study is thus intended to characterize the relationships of ticks and climate, without the restrictive effects of the geographical space. We sought to disentangle the relationships among species and the climate space, as a starting point for further research in the geographical space, which should stand on the dispersion mechanisms as descriptors of the distribution of these taxa as we know today.

## Results

### Taxonomical assemblages of species occupy different regions of climate space

A multivariate analysis of monthly interpolated climate (temperature and rainfall) traits in the Afrotropical region produced 3 main axes, which accounted for the 89.1% of total variability. Axis 1 was loaded by and inversely related to the average annual temperature. Axis 2 was inversely correlated with the range between maximum and minimum temperatures: a large seasonal amplitude in temperature is related to negative values in this axis. Axis 3 was inversely related to total rainfall. Gradients of the first and second PCA axes draw the occurrences of the species, which are restricted to specific portions of such as axes.

**Figure 1:**
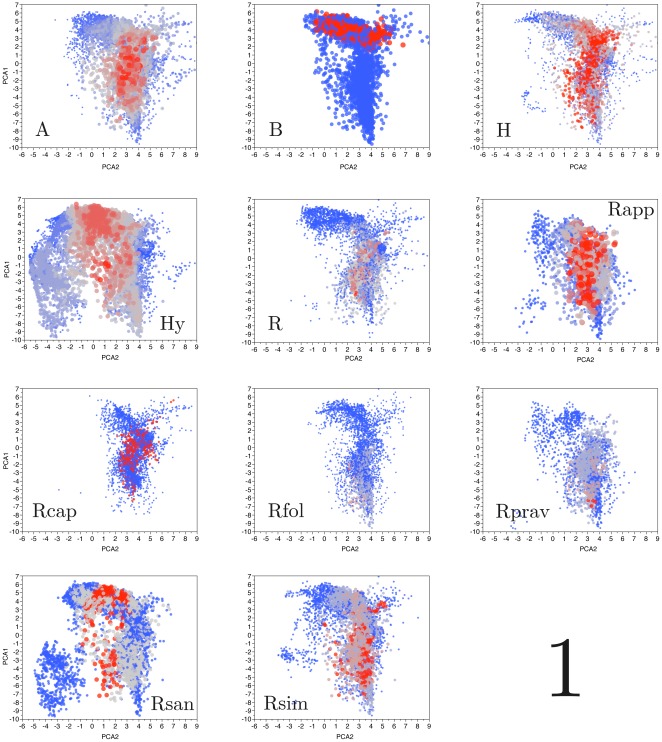
The occurrences of the 72 species of ticks of the Afrotropical region in the climate space. They are clustered according to supraspecific (taxonomic) groups as treated in this study and plotted along the reduced space of the first and second axes of a principal components analysis. The first axis is inversely related to the temperature and the second is inversely related to the thermal variation (temperature variability). Each point represents a unique combination of values in the climate space with positive occurrence. The color and size of each point is proportional to the occurrence values scaled between 0 and 1. Labels are: Genus *Amblyomma* (A), subgenus *Boophilus* (B), genus *Haemaphysalis* (H), genus *Hyalomma* (Hy), species of the genus *Rhipicephalus* (R) not assigned to any morphological group of species, *Rhipicephalus appendiculatus* group of species (Rapp), *R. capensis* group (Rcap), *R. follis* group (Rfol), *R. pravus* group (Rprav), *R. sanguineus* group (Rsan) and *R. simus* group (Rsim). The complete list of species in each taxonomic group or generic arrangement is included in Table 1.

Figure 1 shows the occurrences of 72 tick taxa naturally occurring in the Afrotropical region, grouped along the taxonomic groups of species, as currently recognized by morphological grounds. Most taxonomic groups are tightly attached to a narrow portion of axis 2 (seasonal variability of temperature) while occurring across large portions of axis 1. Examined under such a general scale, the taxonomic groups occur and share similar portions of the climate space. Only the genus *Hyalomma* associates to portions of the climate space with high temperature and a large thermal amplitude, other

**Figure 2:**
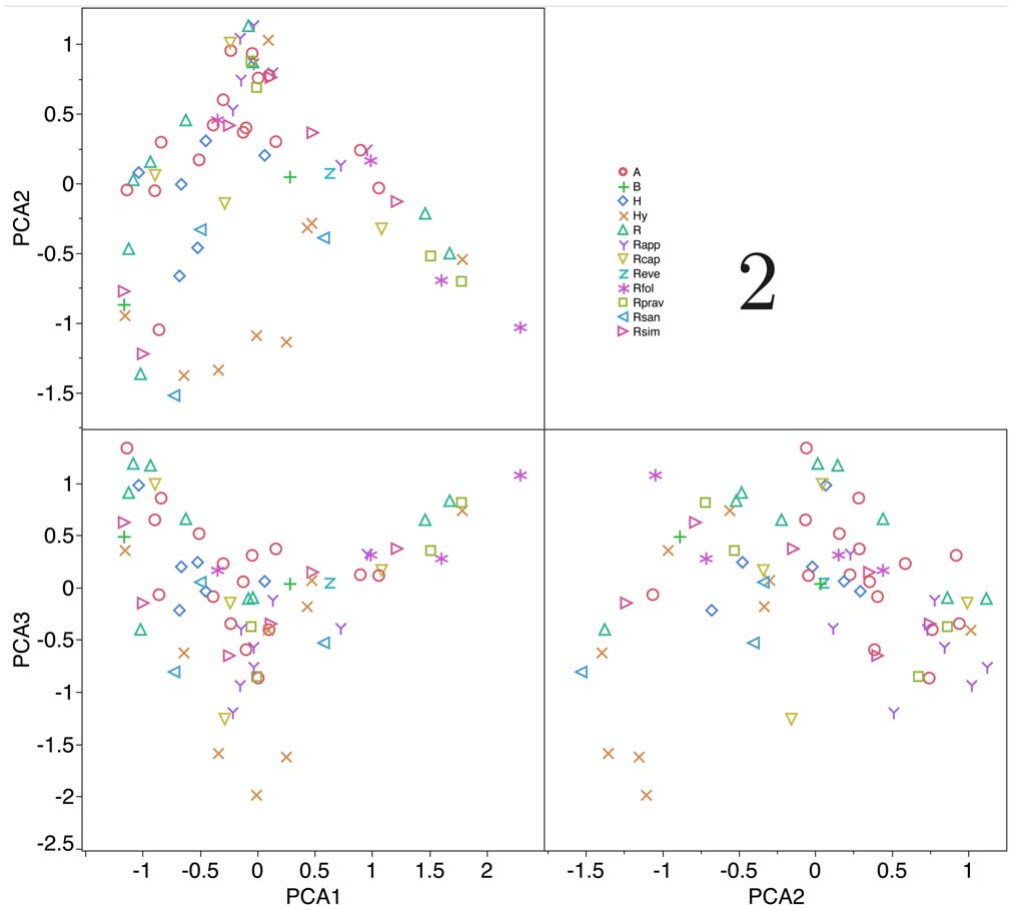
The centroids of the occurrences of the 72 species of ticks in the climate space of the Afrotropical region, with an indication to taxonomic (supraspecific) groups as treated in this study. They are plotted along the combinations of the three axes of a principal components analysis of continuous climate traits. Each taxonomic group has the same symbol, as explained in the legend, and is named according to abbreviations in Table 1 and Figure 1.

Figure 2 shows the centroids of the occupancy of the climate space by the species of ticks along the three gradients derived from PCA. Species sharing the same taxonomic group occur along unrelated portions of the climate space, exception made for most of the species of the genus *Hyalomma*. Systematic similarities among species in the same taxonomic group are not mirrored by the similarities of occurrences along climate gradients. Speciation processes of each morphologically-based, supraspecific group, derived into multiple branches colonizing different portions of the available climate conditions.

**Figure 3:**
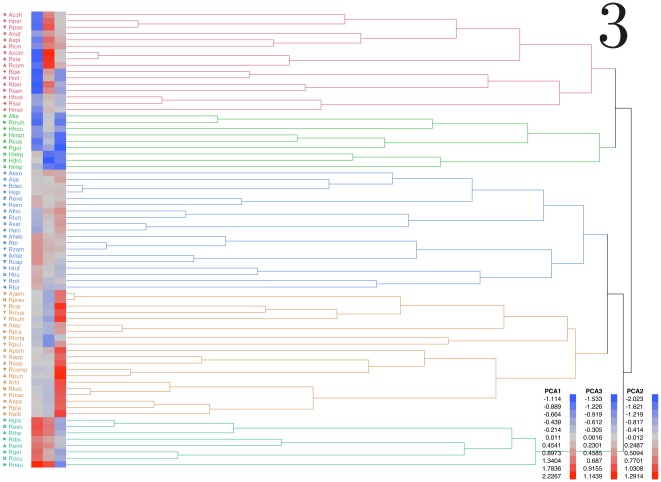
Functional groups of the species of ticks occurring in the Afrotropical region, obtained after a hierarchical clustering of the values of the occurrences of each species at the reduced climate space. The number of clusters explains the most parsimonious solution for the complete clustering of the original data and are marked by colors and consecutive numbers. Each species is named according to the genus and the first three letters of the specific name. Each species is also labeled with the symbol used for such a taxonomic group, as used in Fig. 2. The three blue-red bands near the name of each species are a guide to interpret the mean values of the reduced climate space at which the species occurs. Blue tones relate to low values and red tones relate to high values of each axis.

The occurrence of Afrotropical ticks along gradients of the climate space produced a dendrogram of relationships among the species of ticks (Fig. 3) grouped according their affinities in the climate space and labelled with consecutive numbers. These are the functional groups of species, and the dendrogram confirms the phylogenetic picture of their occurrences across the reduced climate space, different of that based on morphological grounds alone. Groups 1 and 2 occur at warmest portions of the climate envelope, at both regions of low (group 1) and high (group 2) seasonal variability of temperature and variable rainfall patterns. The large group 3 occurs at portions of medium values for each of the three reduced axes of the climate space. Groups 4 and 5 remain associated to progressively colder portions of the reduced climate space. These groups occur in portions of lowest and highest, respectively, seasonal amplitude of temperature.

### Niche equivalency is low among species in taxonomic groups

The Table 1 list the 72 tick species included in the study. The functional groups of species as outlined by the hierarchical clustering of the climate traits at their occurrence points have a small niche equivalency (Table 2). These results were expected since the functional groups segregated according to a maximizing strategy of the climate traits at the specific occurrence points. Calculations thus show the relatively low values of climate niche equivalency between largely separated groups in the dendrogram of Fig. 3. In other words, species belonging to the same functional group share more niche among species in the same group than with species in other functional groups. However, it was an unexpected result that niche equivalency is also low for species within the same taxonomic group (Table 3). Species in some taxonomic groups may be so segregated, as to exhibit values of niche equivalency smaller among them than those computed between different functional groups, whose separation was purposely maximized by the clustering algorithm. Most taxonomic groups in the genus *Rhipicephalus* display low within-group niche equivalency values, other supraspecific taxonomic clusters having a greater niche overlap. Species within each taxonomic lineage in *Rhipicephalus* tend to occur on portions of the climate space more different than expected by random occurrences, therefore maximizing the occupancy of the climate space and avoiding overlaps with other species of the same taxonomic group.

**Table 1:** List of the 72 tick species included in the study.

Taxonomic group	Species	Label
Genus *Amblyomma*	*A. cohaerens*, *A. complanatus*, *A. exornatum*, *A. flavum*, *A. gemma*, *A. hebraeum*, *A. latum*, *A. lepidum*, *A. marmoreum*, *A. nuttalli*, *A. pomposum*, *A. rhinocerinus*, *A. sparnum*, *A splendidum*, *A. tholloni*, *A. variegatum*	A
Subgenus *Boophilus*	*R. decoloratus*, *R. geigyi*	B
Genus *Haemaphysalis*	*H. aciculifer*, *H. hoodi*, *H. houyi*, *H. moreli*, *H. parmata*, *H. spinulosa*	H
Genus *Hyalomma*	*Hy. albiparmatum*, *Hy. impeltatum*, *Hy. impressum*, *Hy. glabrum*, *Hy. nitidum*, *Hy. rufipes*, *Hy. truncatum*, *Hy. dromedarii*, *Hy. aegyptium*	Hy
Genus *Rhipicephalus* (unassigned to groups)	*R. bergeoni*, *R. compositus*, *R. cuspidatus*, *R. mimeticus*, *R. longus*, *R. punctatus*, *R. supertritus*, *R. theileri*, *R. ziemanni*	R
Genus *Rhipicephalus* (*sanguineus* group)	*R. guilhoni*, *R. sulcatus*, *R. turanicus*	Rsan
Genus *Rhipicephalus* (*simus* group)	*R. distinctus*, *R. muhsamae*, *R. planus*, *R. praetextatus*, *R. senegalensis*, *R. simus*	Rsim
Genus *Rhipicephalus* (*follis* group)	*R. gertrudae*, *R. neumanni*, *R. tricuspis*, *R. lunulatus*	Rfol
Genus *Rhipicephalus* (*capensis* group)	*R. compositus*, *R. longus*, *R. pseudolongus*, *R. capensis*	Rcap
Genus *Rhipicephalus* (*appendiculatus* group)	*R. appendiculatus*, *R. carnivoralis*, *R. zambeziensis*, *R. nitens*, *R. humeralis*, *R. muhelensi*, *R. maculatus*, *R. pulchellus*	Rapp
Genus *Rhipicephalus* (*pravus* group)	*R. exophthlamos*, *R. kochi*, *R. pravus*, *R. oculatus*	Rprav
Genus *Rhipicephalus* (*evertsi* group)	*R. evertsi*	Reve

The table displays the taxonomic group as genera, subgenera, or groups of species with taxonomic affinities, the species included in the group and the abbreviation used throughout for that taxonomic group.

**Table 2:** The niche equivalency values of the tick species in the Afrotropical region, as clustered by functional groups computer according a hierarchical clustering algorithm.

	1	2	3	4	5
1	0.378				
2	0.259	0.411			
3	0.237	0.173	0.366		
4	0.227	0.158	0.288	0.395	
5	0.067	0.054	0.238	0.158	0.315

Each value is the climate niche equivalency among the species included in that group (e.g. 1×1) or among the species in different taxonomic clusters (e.g. 1×2). Numbering of the functional groups and the species included in each group follow the consecutively numbered labels of Figure 3.

**Table 3:** The niche equivalency values of the tick species in the Afrotropical region, as clustered by taxonomic groups for the genus *Rhipicephalus* (i.e. groups of species with morphologically similarities) and by generic or subgeneric arrangements for the other species.

	A	B	H	Hy	R	Rapp	Rcap	Reve	Rfol	Rprav	Rsan	Rsim
A	0.321											
B	0.280	0.208										
H	0.372	0.394	0.500									
Hy	0.207	0.222	0.248	0.229								
R	0.232	0.199	0.254	0.156	0.174							
Rapp	0.287	0.195	0.265	0.187	0.168	0.280						
Rcap	0.319	0.230	0.340	0.165	0.237	0.240	0.316					
Reve	0.403	0.432	0.425	0.278	0.239	0.382	0.338	***				
Rfol	0.192	0.189	0.194	0.141	0.166	0.184	0.192	0.323	0.181			
Rprav	0.266	0.176	0.236	0.176	0.216	0.296	0.234	0.378	0.269	0.262		
Rsan	0.280	0.331	0.339	0.293	0.224	0.206	0.210	0.369	0.185	0.208	0.346	
Rsim	0.281	0.270	0.308	0.200	0.187	0.268	0.231	0.359	0.187	0.255	0.251	0.211

Abbreviations follow the denominations of Table 1. Each value is the climate niche equivalency among the species included in that group (e.g. A×A) or among the species in different taxonomic clusters (e.g. A×B). The asterisks mean that only one species of the *R. evertsi* group has been included, and therefore within-group values cannot be calculated.

### Species richness and endemism are associated to specific traits of the climate space

**Figure 4:**
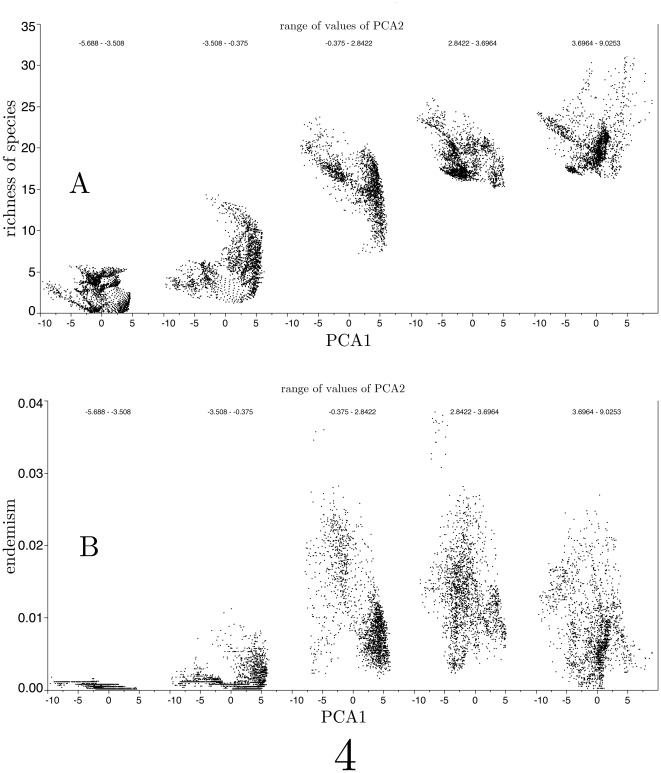
Values of richness of species and endemism in the set of ticks of the Afrotropical region according to the two-first axes of the reduced climate space.

The highest index of species richness is associated to the lowest seasonality (lowest variation of temperature) in temperature values (Fig. 4). Absolute values of temperature have no influence on the index. Even at the portion of the climate gradient where high values of temperature are recorded, species richness remains low if temperature seasonality is high. At medium values of seasonality, richness is higher at higher values of temperature. No specific associations to a rainfall gradient have been noticed (not figured). The pattern of relationships between endemism of species and the climate space is more imprecise, but slightly higher endemism values are consistently associated with higher temperatures and low thermal seasonal variability. At these values of the climate envelope, not only more species exist, but also endemism are concentrated.

## Discussion

The tick fauna associated to the Afrotropical region has long been recognized as to represent an unpaired chance to investigate the many factors giving shape to its current relationships. This framework accounted for biases introduced by spatial resolution and corrects observed occurrence densities for each region considering the availability of climate space. Such a characterization outcomed details about the composition of “functional" groups of species and their phyloclimatic relationships, hypothesizing about the nature of the species-environment relationships. As such, it would be appropriate to construct a general area cladogram (representing a single history of place) based on congruence among the area cladograms derived from phylogenetic analyses of the multiple co-distributed taxa [14].

The Afrotropical fauna of ticks is the result of a diversification of lineages into a variety of taxa currently associated with many biomes [4]. Species occurring in the region show a coherent and thigh association to well defined gradients of climate features, even if examined over the current background of introductions and spreads, driven probably by the movements of domestic animals and other anthropogenic reasons. The scale has no influence in this study because the whole geographic component is translated into the climate space, and a smoothing kernel is applied, thus avoiding any bias related to sampling efforts which might distort the data on the occupancy of specific portions of the climate space [15].

In the case of ticks of the Afrotropical region, groups of taxa have evolved from the primitive pool along different lines of climate pressures and they have occupied different portions of the available climate space. The observed pattern of occurrences along climate gradients suggest that supraspecific assemblages of morphologically similar taxa have a long-standing association with one another and have attained a common pattern of climatically driven subdivision as a result of being subjected to the same environmental history. Specialization is still evident, and species belonging to the same taxonomic group do not tend to occur along similar gradients of climate space. The study of the niche-segregating clusters (i.e. the hierarchical ordination of species according to their occurrences at the climate gradients) shows that functional groups of ticks are dissimilar to the taxonomic groups. Only the genus *Hyalomma* has, in some extent, both a taxonomic and functional identity, most probably because its high specialization towards warmer and drier sites. Every supraspecific group, as morphologically recognized today, has species associated to different portions of the climate niche. In some cases, species within the same taxonomic group share a small portion of climate space. These findings could be interpreted as a strategy by groups of close species evolving from the same genetic pool to exploit the portions of the climate space as much separated as possible. This would minimize competition among species genetically related (same taxonomic group), being restricted by geographical barriers that could effectively operate on the spread patterns over the climate space by restricting movements in the geographical level. The most parsimonious explanation for the different taxonomic groups that exhibit common patterns of climate space subdivision is that they have a shared biogeographic history. In other words, a common set of historical vicariant events has geographically structured a group of ancestrally co-distributed organisms [16]–[18].

The mechanism by which the tick species may colonize and spread into new gradients of the climate space is largely unknown. Support for rapid niche shifts is found in diverse fields of ecology and evolution, its evidence reported in empirical studies of invasive species ecology, phylogenetic analysis and community ecology [19]. Arthropods can expand the range of habitats they occupy and evolve increased dispersal rates in response to climate change and these niche shifts have happened in less than 100 years [18], [19]. However, some studies suggest that niches tend to remain similar in allopatric speciation [20]. Comparisons based on combining phylogenetic methods with empirical data on climate niche characteristics might contribute to a better understanding of niche dynamics by tick species. The characterization of the niches and the combined phylogenetic signal provided by the species in each branch of the cladogram would provide a framework based on underlying ecological and evolutionary mechanisms. For example, a distance matrix that is derived from niche characteristics of species can be compared with a matrix of phylogenetic distances among the species by using a Mantel test or similar randomization. The presence or absence of significant correlation between corresponding elements of the two matrices is interpreted as the presence or absence of phylogenetic signal in the trait [20]. A serious drawback of this methodology is the unavailability of adequate DNA sequences for many species of ticks.

Highest richness of Afrotropical tick species has been found to be correlated with low thermal seasonality and, ranked second, warmer temperatures. Thus, the area of highest species richness is associated with zones where high species richness of birds and mammals is also concentrated [21]. The refuge theory [22] assumes that paleoecologically stable areas would act as arks where local populations of specialized animals and plants survive periods of stress, leading to divergent evolution of the populations remaining in the different refuges. More than 100 hypotheses have been proposed to explain large-scale spatial variation of species richness, but no consensus has yet been reached on the underlying mechanisms [23]. The modeling of the richness of species for Afrotropical ticks has been reported using logistic regression models [24]. However such a pioneering approach was focused on geographical rather than on the climate space, without an explicit effort to understand the drivers of species richness. Although richness of species and endemism are easily observed after a simple visual inspection of the occurrences of the ticks in the climate space, this may be not in all instance the result of an association to specific climate gradients, but split distribution of species along geographical features, like barriers to dispersion restricting host movements, evidenced as areas of local distinctive climate gradients.

The framework built in this paper may be potential drawbacks regarding the ecology of the ticks, the resolution of the climate dataset and the potential bias in reporting of tick collections. The first and the second are strongly linked. The immature stages of some species may have an endophilous behavior, i.e. connected to hosts that live in burrows or protected from hard environmental conditions [25]. There is no way to obtain reliable measurements of climate conditions at a medium resolution under the ground [26]. However, the presence of adult (exophilous) tick stages as reported in the literature review is a reliable indicator of the presence and survival of tick population in the place of reporting, whatever the life history of previous developmental stages is. A bias may anyway occur in species deeply linked to the endophilous way of life, but there are currently no methods to capture their climate niche as occurring in the protected burrow [14]. Unfair reporting because economical or health interest of some tick groups has been already reported [7], [8] and, as a result, the realized distribution in the geographical space would be heterogeneous and unreliable in some sites. However, the applied methodology to the climate space rather than in the spatial one contributes to remove much of the background noise in tick reporting [16]. It is however evident that in such a large area as the Afrotropical region, gaps will exist, which we are unable to correct or even detect.

Species composition is influenced by the combined effects of environmental traits, the portion of the geographical space dominated by those climate gradients and the dispersal properties of the species [27]. It seems plausible that clusters of species, evolving along many lines over wide areas available for the primitive stock, remained linked to these climate gradients and produced an evolutive line, each morphologically similar species evolving along smaller portions of the climate space in the region. The understanding of the distribution of ticks in the Afrotropical region needs a specific geographical-climate analysis to elucidate the currently observed patterns, to underpin the geographical relationships as associated to topographical features, dominant abiotic conditions and patterns of connectivity.

## Methods

We determine climate niche occupancy for a set of tick records in Africa. The framework applies to comparison among any taxonomic, geographic or temporal group of occurrences, and involves the calculation of the density of occurrences and of climate gradients along axes. Those are the result of a multivariate analysis along the climate variables. We then calculated the niche overlap between species together with statistical tests of niche equivalency and the evaluation of richness and endemism of species as associated to coherent portions of the environmental space. Most of the analyses were done in R 2.7.2 [28].

### Sources of records and climate data

Two compilations were primarily used regarding earlier surveys of ticks in the area of interest (since around the year 1950), namely the one reported out in ref. [8] and the one produced by the Integrated Consortium on Ticks and Tick-Borne Diseases (partially available at www.tickbornezoonoses.org). Data from the later were used to update the taxonomic overview in the former and to produce a coherent dataset of records in Africa. The newest available records (after approximately the year 2000) was completed by a systematic search of the peer-reviewed literature between the years 2000 and 2010. The basic criteria included in both searches were the systematic generic or family names of the ticks, as found in the title or the abstract of the paper. Further reading of each reference searched for information about the specific name of the tick, and a strict reference to a site of capture. The site can be defined by its coordinates or by a name providing enough information to locate it in digital gazetteers or regional maps. Records were not included if they referred only to a generic name or if reported from a large administrative division (i.e. province) or if the information regarding the locality was ambiguous (i.e. several localities in the same country sharing the name, without further reference to a province). Literature searches were concluded on 31 October 2010 and all citations meeting our search criteria were reviewed. A total of 10,628 records were included in this study. All the literature data were curated and determinations of taxa replaced with current taxonomical views if necessary, after a consultation to experts. The tick name as appearing in the final dataset is the one agreed in a recent review on the systematics of ticks [29].

Some species were not included in this study because lack of agreement among experts [29] thus being unreliably reported. Other species have been recognized only recently and it is difficult to evaluate the reliability of old records (e.g. *Haemaphysalis leachi* and *H. elliptica*, see ref. [30]). Therefore they were excluded of the final dataset because both species are clearly separated and reported only at the extremes of their distribution ranges, thus providing an unreliable determination in sites where their ranges of distribution overlap. The genus *Ixodes* remains largely unexplored in the Afrotropical region [31]. Its representatives are endophilic species, primarily associated to the microclimatic conditions of the host burrows, therefore precluding an analysis of the macroclimate at their occurrence sites. The complete list of taxa is included in Table 1. Taxonomical groupings for the genus *Rhipicephalus* adhered to the views summarized in ref. [32]. These are assumed clusters of species (taxonomic groups) with phylogenetic affinities based only on morphology, since DNA sequences are unavailable for most of these species.

Climate data were obtained from WorldClim [9] a set of monthly climate features covering the world. The area covering the Afrotropical region was selected from the dataset, clipped with a mask of the terrestrial contour, and used as source for further analysis. We used monthly average temperatures and monthly rainfall values, which were subjected to a principal components analysis to reduce the number of variables without loosing biological information.

### Climate space and occurrence density

We consider the first three axes of the principal components analysis of the monthly climate variables as definition of the climate space of the ticks [15]. The climate space is thus bounded by the maximum and maximum climate values found across the entire study region. In principle, climate space occupancy by ticks can consider greater dimensionality, but in practice increased dimensionality brings greater challenges for interpretation. The environmental space is divided into a grid of *r*×*r* cells of a three dimensional volume (which we call here the climate “space") each cell being a unique vector of climate conditions present at one or more sites of the geographic space.

The occurrences of each species in each unique cell of the grid was used to map the occurrence of the ticks in the climate space. The number of occurrences of a tick species is dependent on sampling strategy, and a dataset may not entirely reflect the actual distribution of a species. We adhered to published methods [16] and applied a standard Gaussian kernel to determine the smoothed density of occurrences along the environmental axes. The smoothed density of occurrence *O_ijk_* for each cell is calculated as:
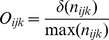
where 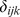 is the kernel density estimation of the number of occurrences of the tick species at sites with the unique climate 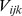 and 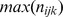 is the observed maximum number of occurrences in any one cell. The smoothed density of available climate conditions 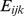 is calculated as:
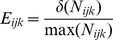
where 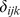 is the is the number of sites with the climate and 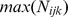 is the number of cells of the grid with the most common climate conditions. 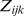 is the occupancy of the climate 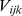 by each individual taxa, and calculated as
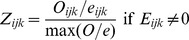
and
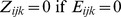
where 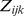 ranges between 0 and 1 allowing an unbiased comparison of occurrence densities occurring in ranges where climate space is not equally available [16]. Thus, each unique vector of climate conditions is loaded with the smoothed density of occurrences.

Most of the calculations were done in R 2.7.2 [28]. We plotted the complete set of smoothed occurrences for each species, both separately and by taxonomic groups, as included in Table 1. We used the centroids of the distribution of each species in the climate space to unambiguously define its position. We performed a hierarchical clustering by the Ward's method, as available in JMP version 9, on the occurrences of each species according to the three climate axes. Such a procedure is simply a niche ordering model that clusters the species of ticks into the groups according to the values of the climate space at each point of occurrence. It is a dendrogram displaying the functional affinities of the species.

### Comparing climate niches

Niche overlap between any two species involves three steps: (1) calculation of the density of occurrences and of climate factors along the axes of the multivariate analysis before (2) measurement of niche overlap along the gradients of this multivariate analysis and (3) statistical tests of niche equivalency [16], [33].

The comparison of 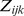 between two entities can be used to calculate niche overlap using the D metric [32].
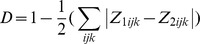
where 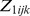 is entity 1 occupancy and 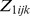 is entity 2 occupancy. This metric varies between 0 (no overlap) and 1 (complete overlap). It has been reported [16] that the use of a kernel density function is critical for an unbiased estimate of D. When no kernel density function is applied, the calculated overlap depends on the resolution r chosen for the gridded environmental space. Using smoothed densities from a kernel density function ensures that the measured overlap is independent of the resolution of the grid [16].

We built from the methodology previously described [33], [34] to perform niche equivalency tests. The niche equivalency test determines whether niches of two entities in two geographical ranges are equivalent (i.e. whether the niche overlap is constant when randomly reallocating the occurrences of both entities among the two ranges). All occurrences are pooled and randomly split into two datasets, maintaining the number of occurrences as in the original datasets, and the niche overlap statistic D is calculated. This process is repeated 100 times (to ensure that the null hypothesis can be rejected with high confidence) and a histogram of simulated values is constructed. If the observed value of D falls within the density of 95% of the simulated values, the null hypothesis of niche equivalency cannot be rejected.

### Species richness and endemism

Measures of species richness are statistical measures. There is no consensus on best estimators of species richness. In this study we sought to investigate how a range of climate factors drive taxonomic diversity in the ticks reported in the Afrotropical region. More specifically, we tested whether regions with a given gradient in the axes of climate space may harbor different diversity. We purposely remain general in scope to explore how hypotheses can be extrapolated to the taxonomic diversity of a group of ectoparasites.

We computed species richness weighting the number of smoothed occurrences at each vector of the climate space with the total values of occurrences. To compute species endemism in the climate space, we estimated the number of species present at every grid squares of the three dimensional climate space. Each species is down-weighted by the number of grid squares in which it occurs, and the index is then the sum of the range-down-weighted species values for each grid. The down-weighting was calculated by dividing each grid-occurrence by the total number of grids in which that species occurs. Thus a species restricted to a single grid would be scored as ‘1’ for that grid, and ‘0’ for all other grids; a species found in two grids, would be scored as ‘0.5’ for each of the two grids, and ‘0’ for all other grids; a species found in three grids would be scored as ‘0.333’ for each of the grids, and ‘0’ for all other grids, etc.
